# A Bright Horizon: Immunotherapy for Pediatric T-Cell Malignancies

**DOI:** 10.3390/ijms23158600

**Published:** 2022-08-02

**Authors:** Haley Newman, David T. Teachey

**Affiliations:** 1 Division of Oncology, The Children’s Hospital of Philadelphia, Philadelphia, PA 19104, USA; teacheyd@chop.edu; 2 Perelman School of Medicine, University of Pennsylvania, Philadelphia, PA 19104, USA

**Keywords:** T-cell acute lymphoblastic leukemia, anaplastic large cell lymphoma, pediatric cancer, immunotherapy, CAR T-cell therapy

## Abstract

Immunotherapy has transformed the treatment of hematologic malignancies in the past two decades. The treatment of acute lymphoblastic leukemia (ALL), in particular, has been highly impacted by multiple novel immunotherapies. For pediatric patients with T-cell malignancies, translating immunotherapies has proved more challenging due to the complexities of fratricide, risk of product contamination with malignant cells, and concerns over T-cell aplasia. Despite these hurdles, many creative and promising strategies are on the horizon. We review challenges in the development of immunotherapy for T-cell malignancies, strategies to overcome these challenges, as well as therapies currently being investigated and starting to reach the clinic. Immunotherapy will hopefully successfully treat patients with relapsed and refractory T-cell malignancies and may someday be incorporated in up-front protocols in order to prevent relapses.

## 1. Introduction

Immunotherapy has transformed the treatment of hematologic malignancies in the past two decades. The treatment of acute lymphoblastic leukemia (ALL), in particular, has been highly impacted by multiple novel immunotherapies. Children with relapsed or refractory B-cell acute lymphoblastic leukemia (B-ALL) who were once considered incurable now have a multitude of options for salvage therapy from targeted antibodies to chimeric antigen receptor T-cells. For pediatric patients with T-cell acute lymphoblastic leukemia (T-ALL), however, translating immunotherapies has not been as swift or straightforward due to the complexities of fratricide, risk of product contamination with malignant cells, and the potential toxicity of T-cell aplasia. Despite these hurdles, many creative and promising strategies are on the horizon for T-ALL and are now starting to reach the clinic. 

Immunotherapy harnesses the body’s own immune system to engage in cancer-directed cell killing. The first immunotherapy agents clinically available were unconjugated monoclonal antibodies (mAb) which work either by complement-dependent cytotoxicity (CDC) or antibody-dependent cellular toxicity (ADCC). In CDC, mAbs bind the cell surface and activate the complement cascade, leading to formation of the membrane attack complex and subsequent cell lysis [1]. In ADCC, antibodies bind both antigens on tumor cells and Fc receptors on the surface of immune effector cells, engaging effector cell targeting [2]. Rituximab is the hallmark example of an ADCC: a human/murine chimeric anti-CD20 monoclonal antibody, it binds CD20 on target cells and the Fcγ receptors of immune effector cells, triggering release of proteins and proteases leading to target cell death [3]. Rituximab is now part of standard treatment regimens for B-cell non-Hodgkin lymphomas in adults and children [4], as well as in adults with B-ALL [5], prolonging survival of many patients with CD20-positive disease [6]. 

While monoclonal antibodies have demonstrated marked benefit in some lymphomas, overall improvements with unconjugated mAbs in hematologic malignancies have been modest [2]. Thus, new waves of immunotherapies followed suit. After basic monoclonal antibodies, antibody-drug conjugates (ADC) and Bispecific T-cell engagers (BiTE) were developed. ADCs consist of recombinant monoclonal antibodies covalently bound to cytotoxic chemicals via synthetic linkers [7]. Examples of ADCs in clinical practice include gemtuzumab ozogamicin (anti-CD33 mAb conjugated to calicheamicin antitumor antibiotic), which is used in pediatrics in combination therapy for CD33+ AML, Brentuximab vedotin (anti-CD30 antibody conjugated to an anti-mitotic small molecule) used in CD30+ lymphomas [8,9], and inotuzumab ozogamicin (anti-CD22 antibody conjugated to calicheamicin) for CD22+ ALL [10]. BiTEs work by simultaneously binding a marker on a malignant cell and an endogenous T-cell, enhancing activated T-cell engagement by triggering cytotoxic granule fusion, cytokine release and T-cell proliferation [11]. The most ubiquitous BiTE in clinical practice for hematologic malignancies is blinatumomab, a bispecific monoclonal antibody construct which simultaneously binds CD3-positive cytotoxic T-cells and CD19-positive B-cells to activate T-cell recognition and killing of CD19 positive blasts [12,13]. 

Beyond antibody-based immunotherapies, adoptive cell therapies (ACT) emerged with rapid advancement. The most widespread ACT is chimeric antigen receptor (CAR) T-cells: T-cells which have been genetically modified to target specific antigens expressed on cancer cells. CAR T-cell therapy has been highly successful for *r*/*r* B-cell malignancies, and two CAR T-cell products targeting CD19 are FDA approved for ALL and lymphoma: Tisaglenlecleucel and Axicabtagene ciloleucel [14,15,16]. Nonetheless, approximately 50% of pediatric patients with B-ALL treated with CD19-directed CAR T-cells eventually relapse [17]—an important area of investigation for CAR T-cell improvement. In addition to CAR T-cells, Natural Killer (NK) cell CARs and macrophage-based CARs are also being explored and are of particular interest for hematologic malignancies [18,19]. 

T-cell malignancies are diseases arising from precursor or mature T-cells. In children and young adults, they can be broadly grouped as (a) T-ALL and T-cell lymphoblastic lymphoma (T-LBL), and (b) peripheral T-cell lymphomas (PTCL). T-ALL is the most common T-cell malignancy in children, accounting for approximately 15% of all pediatric ALL cases [20]. T-LBL makes up about one third of all pediatric cases of non-Hodgkin lymphoma (NHL) [21]. 

T-ALL and T-LBL are generally considered on a spectrum of the same disease, with nearly identical immunophenotypic changes. The major difference is a greater propensity of T-ALL to invade extra-lymphatic spaces, including the central nervous system (CNS). The diseases are differentiated based on degree of bone marrow involvement, with <25% of marrow involvement considered T-LBL and >25% considered T-ALL—although, notably, T-ALL may still present with lymph node involvement and T-LBL patients often have low level marrow involvement (5–25%) [22,23]. 

Over time, the therapy for T-ALL and T-LBL has been harmonized as T-LBL treatment transitioned from lymphoma-like therapy to leukemia-like therapy with multiple studies demonstrating superior efficacy with leukemia-like treatment [24,25]. In the most recent Children’s Oncology Group (COG) clinical trial (AALL1231), however, patients with T-LBL had significantly improved survival outcomes with the addition of the proteasome inhibitor bortezomib, whereas outcomes in T-ALL were equivocal [26], suggesting that there may be biological differences that have yet to be elucidated. 

PTCLs are a heterogenous group of NHLs that originate outside of the thymus and bone marrow. The most common PTCL in children is anaplastic large cell lymphoma (ALCL), and more than 90% of cases have aberrant expression of ALK fusion proteins [27]. 

Despite improved outcomes for de novo T-ALL, the prognosis for patients with relapsed T-ALL is dismal—today, less than 30% of patients who relapse will be cured [28], and outcomes for relapsed T-LBL are even worse [29]. Thus, novel treatments are urgently needed to prevent relapse and treat children and adolescents with relapsed T-cell malignancies. Integration of creative immunotherapy strategies in preclinical models and early phase clinical trials are described in this review. First, we describe challenges of immunotherapy for T-cell malignancies, then report clinically relevant treatments being investigated, and finally summarize the landscape of immunotherapy for T-cell malignancies with consideration of what may come next. 

The main targets under investigation for T-cell malignancies summarized in this review include: CD1a, CD4, CD5, CD7, CD25, CD30, CD37, CD38, CD52, CD194, IL7ra, hTERT, PD1, CTLA4, and TRBC1. These target antigens and open clinical trials are summarized in Table 1.

## 2. Challenges of Immunotherapy in T-Cell Malignancies

Immunotherapy has transformed treatment of B-cell malignancies in part because of unique targeting properties: targetable cell surface proteins (CD19, CD22) are commonly expressed in the cancer cells in most patients and are generally specific to B-cells with minimal (or tolerable) off-target expression in healthy tissues. Moreover, B-cell aplasia, the ubiquitous immunologic side-effect of CD19 targeting, can be successfully managed with intravenous immunoglobulin (IVIG) replacement. 

Finding the right target for T-cell malignancies has proved more challenging. Commonly expressed markers on T-ALL blasts, such as CD3, CD5 and CD7, are also prominent in healthy T-cells. Unlike B-cell aplasia which can be treated with immunoglobulin replacement, T-cell aplasia is a potentially life-threatening immunodeficiency that can only be managed with bone marrow transplant. Thus, in targeting T-cell markers, extra steps are required in some cases to mitigate killing of healthy T-cells—or immunotherapy is considered a bridge to bone marrow transplant. An additional challenge is that some targets are not universally expressed in all T-cell malignancies; for example, CD123 is commonly expressed in early T-cell precursor (ETP) ALL but rarely in more mature T-cell malignancies, which limits the scope of its use to a sub-population of T-ALL.

Here, we summarize additional complexities of T-cell directed immunotherapy, including fratricide, product contamination, immunosuppression, graft versus host disease (GVHD), and general toxicities. 

### 2.1. Fratricide

A major challenge in the use of CAR T-cells for T-cell malignancies is the issue of fratricide: self-killing due to shared antigenicity of the target and the CAR T-cell itself. Notably, fratricide seems to be a greater problem for some T-cell antigens than others. For example, CD7 CAR targeting seems highly prone to fratricide and requires genetic manipulation of the CAR T-cell to dampen CD7 expression and allow for CAR-T proliferation. Alternatively, CD5 targeting seems to have less hinderance with fratricidal killing, and thus CAR-T targeting CD5 has demonstrated success even without genetic manipulation—although this seems to depend in part on the costimulatory domain. One preclinical study of a CD5-CAR T-cell with CD28 costimulatory domain led to surface downregulation of CD5 in CAR T-cells with minimal fratricide, good expansion, and significant in vitro cytotoxicity against T-ALL cell lines [30]; however, with the use of 4-1BB as the costimulatory domain, the CD5-CAR construct demonstrated more fratricide [31]. This was circumvented through a “Tet-off” system in which the CD5-CAR T-cells were first expanded in the presence of doxycycline in vitro which inhibits CAR expression, and subsequently anti-CD5 CAR T-cell expression was restored in vivo in the absence of doxycycline [31]. In an AML CAR therapy targeting GRP78, Hebbar and colleagues demonstrated that using dastatinib during GRP78-CAR T-cell manufacturing blocked CAR signaling and GRP78 cell surface expression, improving efficacy in in vitro and mouse models [32]. 

Other modalities to avoid fratricide include genome editing with CRISPR-Cas9 and cytosine base editing (CBE) techniques, as well as protein expression blockers (PEBL). Details on the use of these techniques are described in the CAR T-cell therapy “Off the Shelf” section of this review. 

### 2.2. Product Contamination

Circulating malignant T-cells can be found in the bloodstream of patients with T-ALL and have phenotypic and functional properties mirroring healthy T-cells. Thus, there is a risk that malignant T-cells may be harvested, transduced, and re-infused as “CAR tumor T-cells”. Ruella and colleagues described an accidental CAR construct of leukemic B-ALL cells which led to a case of CAR-transduced B cell leukemia relapse [33]. The risk of this occurring in T-ALL is even greater than in B-ALL, as purifying apheresis products from circulating tumor T-cells is more challenging. Alternative approaches of interest which obviate this challenge include allogeneic “off-the shelf” CAR T-cells from healthy donors, or NK-cell derived cell products. 

### 2.3. Immunosuppression: T-Cell Aplasia

In the case of CD-19 directed CAR T-cells for B-cell malignancies, off-target effects cause prolonged B-cell aplasia, which can be managed with long-term immunoglobulin replacement. Unfortunately, T-cell aplasia poses a greater challenge as profound loss of T-cells in children would mirror Severe Combined Immune Deficiency, and there are no available T-cell replacement strategies. Thus, it would be expected that T-cell directed immunotherapies could cause severe T-cell aplasia, which necessitates subsequent hematopoietic stem cell transplant (HSCT). Surprisingly, however, data from multiple early phase trials have found recovery of nonmalignant T-cells with negative expression of the targeted antigen, which may, in some instances, mitigate this challenge [30,34]. 

Several approaches to this dilemma are also being investigated. One avenue is delivery of CAR mRNA through electroporation, a non-viral vector method that allows for fine-tuning transfection protocols. Because mRNA expression is transient, multiple infusions are required. In a preclinical model, mRNA electroporation of T-cells for CD19-CAR expression demonstrated prolonged survival of a xenograft mouse model; however, unsurprisingly, as mRNA levels decreased tumor burden increased [35]. To our knowledge this technique has not yet been explored for T-ALL. 

Novel approaches have emerged to control CAR T-cell duration to ameliorate immunosuppression, off-tumor activity, and side effects, including pre-programmed suicide genes and safety switches. Eyquem and colleagues also generated a CD19-specific CAR to the T-cell α constant (*TRAC*) locus, which led to uniform CAR expression in peripheral blood T-cells and enhanced T-cell potency. Furthermore, in a mouse model, these CAR T-cells demonstrated more effective killing of leukemic blasts than conventional CAR T-cells. This may be another model for T-ALL-directed immunotherapy in the future [36]

These strategies are summarized in detail in by Brandt and colleagues in “Emerging Approaches for Regulation and Control of CAR T cells: A Mini Review” [37]. 

### 2.4. Graft versus Host Disease 

GVHD is an adverse immunologic condition that can occur following allogeneic stem cell transplant when donor-derived T-cells attack host tissues. Acute and chronic GVHD are triggered by magnified immunologic responses causing a range of symptoms from severe rashes to chronic diarrhea with high morbidity and mortality. 

In an effort to mitigate challenges of host T-cell fitness, manufacturing and fratricide, there has been growing interest in allogeneic (“off the shelf”) CAR T-cells. A concern of allogeneic CARs, however, is subsequent risk of GVHD in a mechanism similar to that of GVHD post allogeneic transplant with donor T-cells seeing host tissues as foreign, triggering immune attack. There are ongoing efforts to mediate this process with genome editing and posttranslational processing. Gomes-Silva and colleagues demonstrated that using CRISPR/Cas9 technology, deletion of the T-cell receptor (TCR) alpha chain on CD7 directed CAR T-cells prevented GVHD through blocked T-cell signaling [38]. Cooper and colleagues used a similar mechanism to create CD7 CAR T-cells with a disrupted TCR alpha chain which successfully killed human T-ALL cell lines in vitro and in vivo without evidence of xenograft model GVHD [39]. 

There may be other mechanisms to prevent GVHD with allogenic CARs, as well as for autologous CARs that could disrupt the host T-cell milieu and prevent CAR T-cell driven GVHD. This area warrants further investigation. 

### 2.5. Immunotherapy Side Effects

The most common toxicities associated with CD19-directed CAR T-cell therapy are cytokine release syndrome (CRS) and immune effector cell associated neurotoxicity syndrome (ICANS) [40]. CRS is a disorder of fever and inflammation caused by the secondarily activated immune cells. After CAR T-cells ligate to ALL blasts they undergo activation and proliferation, which subsequently activates other immune cells leading to release of pro-inflammatory cytokines [41,42]. In most patients, CRS is mild and reversible; however, in some cases it can cause refractory shock, multiorgan system failure, and a clinical and cytokine presentation mimicking hemophagocytic lymphohistiocytosis [42,43]. ICANS is a less well understood type of neurotoxicity which can present with a constellation of symptoms post CAR T-cell infusion, including: encephalopathy, tremor, delirium, aphasia, cerebral edema and death. The exact mechanism for severe ICANS is unclear but may relate to increased blood–brain barrier permeability and high concentrations of pro-inflammatory cytokines within the CNS [44]. 

CRS and ICANS are not exclusive to CD19-directed CAR T-cells; they also occur with other T-cell engaging therapies, including BiTEs such as blinatumomab [45,46], and with other CAR therapies. CRS and ICANS will likely be seen with T-cell directed immunotherapies; however, it remains to be seen if the severity, frequency, kinetics and biology mirror the B-ALL experience.

## 3. Immunotherapy for T-ALL

### 3.1. Antibodies

#### 3.1.1. CD38: Daratumumab, Isatuximab

CD38 is a catalytic membrane protein which hydrolyzes NAD+ and is important for intracellular calcium metabolism [47]. It is expressed in relatively low levels on hematopoietic progenitor cells, NK cells, monocytes, and activated T- and B-cells [48], as well as non-hematopoietic tissues, including endothelial cells [49], neurons [50], and pancreatic beta cells [51]. CD38 is highly expressed in multiple myeloma and some ALL and AML blasts [52], with findings of persistent expression even after chemotherapy exposure [53]. Given its common expression in multiple myeloma and T-cell malignancies and minimal expression on pluripotent hematopoietic stem cells, CD38 has emerged as an attractive drug target [54]. 

Daratumumab is a humanized anti-CD38 IgG1k monoclonal antibody with FDA approval for treatment of multiple myeloma and many ongoing studies in T-cell malignancies. Daratumumab induces apoptosis by cell-mediated cytotoxicity, complement activation, and phagocytosis. Preclinical models demonstrated that daratumumab had significant activity in T-ALL xenografts as a single agent and in combination with traditional chemotherapy [55,56]. In a recent phase II clinical trial, daratumumab was investigated in combination with chemotherapy for children and young adults with *r*/*r* T-ALL or T-LBL. It was administered with the Berlin-Frankfurt-Münster (BFM) backbone induction and consolidation chemotherapy (vincristine, prednisone, doxorubicin, pegasparaginase, cyclophosphamide, 6MP, methotrexate). Among 22 response-evaluable T-ALL patients, 10 patients (41.7%) achieved CR at end of induction and 63.6% of subjects were in CR or CRi (complete remission with incomplete count recovery). The overall response rate by the end of consolidation (cycle 2) was 90.9% (*n* = 20) [57]. The trial was also successful in getting *r*/*r* T-ALL and T-LBL patients to HSCT: 15 of 24, 62.5%. Comparing this trial to the most recent Children’s Oncology Group (COG) clinical trial with a similar cohort (AALL07P1), the overall response rate was similar after cycle 1 (63.6% vs. 68%), as was 12-month event free survival (EFS; 49.4% vs. 45%) and 12-month overall survival (OS; 57.9% vs. 59%), yet the addition of daratumumab improved successful bridging of patients to transplant (62.5% vs. 45%). AALL07P1 used the same cycle 1 backbone except included two more doses of pegaspargase and four doses of bortezomib. There have also been several anecdotal case reports published using daratumumab with varying degrees of success in highly refractory patients [58,59,60]. 

In 2015, daratumumab was FDA approved for the treatment of multiple myeloma and is now widely used in relapses [61]. Notably, it is associated with high rates of infusion-related reactions (at first infusion only), with symptoms of rhinorrhea, cough, shortness of breath, bronchospasm, chills and nausea [62]. In two phase three trials for *r*/*r* multiple myeloma, infusion-related reactions occurred in 45% and 48% of patients [63,64]. Reactions can be mitigated with slower infusion rates and pre-medication prophylaxis (diphenhydramine, methylprednisolone or dexamethasone, and acetaminophen) [61,62]. While preliminary data have shown reassuring safety profiles in pediatric T-cell malignancies, monitoring for severe infusion reactions remains important.

Daratumumab is slower acting than other pharmacologic agents used in the up-front setting, and thus determination of treatment response may require adjustment of standard timelines. As an unconjugated monoclonal antibody, it is unlikely to be efficacious as a sole treatment; however, it may be more promising in conjunction with other therapies. In a preclinical xenograft T-ALL model, Müller and colleagues recently demonstrated that co-targeting of CD47 and CD38 with anti-CD47 monoclonal antibodies plus daratumumab in combination significantly enhanced in vitro phagocytosis and prolonged survival as compared with single agent treatment [65]. 

Additional studies of combination therapy with daratumumab are warranted. There may also be a role for daratumumab as a bridging agent for persistent low-level disease before hematopoietic stem cell transplant (HSCT), re-emergence of MRD post-transplant, and as a consolidative agent in MRD-negative patients post-transplant.

There are two open clinical trials investigating daratumumab for *r*/*r* T-ALL and T-LBL, including an investigation for T-ALL patients in morphologic remission but with persistent or relapsed MRD by flow cytometry (NCT05289687), and the aforementioned phase II trial which is still active (NCT03384654). 

Isatuximab is also a monoclonal antibody directed to a different epitope on CD38 which induces apoptosis through multiple mechanisms, including ADCC and complement fixation [66]. A recent phase II clinical trial studied the efficacy of Isatuximab monotherapy for relapsed or refractory T-ALL or T-LBL in older adults (NCT02999633). Unfortunately, no patients achieved complete response and most patients had disease progression [67]. 

In multiple myeloma, the kinetics of therapy with anti-CD38 monoclonal antibodies was in the order of weeks for evidence of response. T-ALL is an aggressive malignancy, thus slow antibody targeting with a single agent may be inadequate. An alternative approach is antibody in conjunction with cytotoxic chemotherapy, which is being studied in an open phase II trial of isatuximab in combination with chemotherapy for pediatric patients with relapsed or refractory ALL or AML (NCT03860844).

#### 3.1.2. CD52: Alemtuzumab

CD52 (Campath-1 antigen) is a small surface glycoprotein expressed on lymphocytes, monocytes, dendritic cells and in the male genital tract (epididymis) and on the surface of mature sperm [68]. Its function is not well defined; however, experiments with anti-CD52 antibodies have suggested that CD52 is important for lymphocyte trans-endothelial migration and may influence co-stimulation of CD4+ T-cells [69]. CD52 expression on many malignant lymphoid cells but low expression on hematopoietic progenitor cells has also sparked interest in targeting [70], although with less success than CD38 targeting thus far. 

Alemtuzumab is a humanized anti-CD52 monoclonal antibody. A phase II study of Alemtuzumab in adult patients with HTLV-1-Associated T-ALL and T-LBL demonstrated acceptable toxicity, but short response duration with a medial progression-free survival time of 2 months and median overall survival of 5.9 months [71]. Results from a phase I trial of alemtuzumab in combination with cytotoxic chemotherapy (CALGB 10103) were slightly more promising with reduction in MRD; however, there was a high incidence of serious viral infections [72]. A phase II study of alemtuzumab in children with *r*/*r* ALL found only 1 of 13 patients with a complete response and 4 with stable disease. Additionally, two patients experienced dose limiting toxicity with pain and allergic reaction/hypersensitivity; thus, it was not recommended that single agent alemtuzumab be explored further in children with *r*/*r* ALL [73]. 

There is one open phase I clinical trial for adults with T-cell promyelocytic leukemia (TPML) exploring combination therapy of alemtuzumab with itacitinib (Jak1 inhibitor; NCT03989466). 

#### 3.1.3. CD25: Basiliximab 

CD25, also termed IL2RA, is the alpha chain component of the interleukin 2 receptor, which homodimerizes with IL2RB (beta chain). CD25 is important for T-cell proliferation, cell death, and actions of regulatory and effector T-cells. CD25 is present on activated T-cells and B-cells, as well as low level expression in thymocytes [74]. Clinically, it is heterogeneously expressed in ALL and lymphomas. 

Basiliximab is a monoclonal antibody targeting CD25, which was previously used as immunosuppression for solid organ transplant recipients [75]. In an isolated case report, a 5-year-old girl with T-ALL developed progressive eruption of crusted papules and plaques consistent with febrile ulceronecrotic Mucha-Habermann disease (FUMHD). Given concern for superinfection, her chemotherapy could not be intensified. The addition of basiliximab improved her skin findings and allowed for intensification of therapy and bone marrow transplant. Given this case, there may be opportunity for the use of anti-CD25 agents in combination therapy for T-ALL [76]. To our knowledge, there are no open trials using this drug for T-cell malignancies. 

#### 3.1.4. IL7rα

The interleukin-7 (IL7) receptor, a heterodimer expressed on the surface of lymphocytes, is composed of the IL7 receptor-α (IL7rα) subunit and the common-γ chain receptor. IL7 and IL7r are critical for T-cell development and differentiation; in fact, their absence leads to severe immunodeficiency. Alternatively, excess IL7 or IL7 receptor signaling can drive development of lymphoid leukemia [77]. Hixon and colleagues found that murine monoclonal antibodies targeting the human IL-7rα chain mediated cytotoxicity against patient-derived xenograft (PDX) T-ALL cells [78]. Akapeddi and colleagues further demonstrated that an anti-IL7rα antibody reduced tumor burden and promoted survival in PDX models [79]. These findings require additional exploration for understanding of efficacy and safety in the clinical context.

#### 3.1.5. CD194/CCR4: Mogamulizumab

Chemokines and chemokine receptors harmonize immune cell movement and homing within body tissues. CC-chemokine receptor 4 (CCR4) is a receptor for two chemokine ligands (CCL17 and CCL22) and is mainly expressed on Type 2 helper T-cells (Th2) cells and regulatory T-cells (Treg) cells, inducing homing of leukocytes to sites of inflammation. CCR4 is often highly expressed in mature T-cell neoplasms [80].

Mogamulizumab (Mog) is a humanized antibody targeting CCR4. In the United States it is FDA approved for treatment of relapsed or refractory Mycosis Fungoides and Sezary syndrome. In Japan, it is also approved for CCR4+ adult T-cell leukemia. Shichijo et al. demonstrated improved four-year OS with mogamulizumab plus chemotherapy as compared with chemotherapy alone for transplant ineligible patients with adult T-ALL. [81]. There is one open clinical trial examining mogamulizumab in combination with third-party NK cells for adults with *r*/*r* T-ALL or T-LBL (NCT04848064). Of note, in patients with cutaneous T-cell lymphoma treated with mogamulizumab, resistance was frequently associated with loss of CCR4 expression; [82] thus, this escape mechanism should be examined with its use with other diseases. 

#### 3.1.6. CD1a

CD1a is a glycoprotein expressed on transient thymocyte populations and Langerhans cells. It is thought to play a role in lipid antigen presentation and in host immunity [83,84]. In patients with cortical-derived T-ALL, about 40% express CD1a; however, it is not typically expressed in more mature healthy T-cells or mature T-cell malignancies. Recently, Riillo and colleagues developed a BiTE simultaneously targeting CD1a and CD3e(epsilon), which induces activation, proliferation, and cytokine release by T-cells against CD1a+ T-ALL cells in vitro [85]. To our knowledge, there have not been any in vivo studies targeting CD1a. 

### 3.2. CAR T-Cell Therapy

#### 3.2.1. CD7 CAR

CD7 is a glycoprotein which is part of the immunoglobulin superfamily cell adhesion molecules, a diverse group of receptors which are essential to both humoral and cell-mediated immune reactions. CD7 has been shown to have costimulatory activity and induce tyrosine and lipid kinases [86]. CD7 is highly expressed in T-ALL and in some PTCLs. Normal expression is mainly restricted to T-cells and NK cells, reducing off-target toxicity. 

Pan and colleagues recently published results from the first phase I trial of anti-CD7 CAR T-cells in children and adults with *r*/*r* T-ALL. Patients who had previously received HSCT (with persistent donor chimerism) received T-cells from their prior donors, while patients without prior HSCT received CAR T-cells sourced from donors whose stem cells would subsequently be used for HSCT about 30 days post CAR T-cell therapy if remission was achieved. Remarkably, 19 of 20 patients responded to treatment, and 18 had complete morphologic remission. CAR T-cell expansion was detected in both blood and CSF of many patients. To decrease risk of fratricide, the CAR construct was genetically modified using Intrablock technology to prevent CD7 expression on the CAR cell surface. All patients demonstrated depletion of normal CD7+ lymphocyte populations, while nonmalignant CD7− T-cell levels rose. While these CD7− T-cells had less T-cell receptor diversity than T-cells from healthy individuals, they were still able to react in response to fungal stimuli and with exposure to viral peptides. Although response rates were high in this cohort and results are promising, median follow-up was short (6.3 months, range 4–9.2 months). Longer follow up assessment is still warranted to determine durability of remission [34]. 

The utilization of donor-derived T-cells for CAR T-cell manufacturing (pre- or post-HSCT) is a novel approach. Other groups have created autologous anti-CD7 CAR T-cells with genomic editing, or allogeneic modified CAR constructs. CRISPR-Cas9 editing of CD7 has been utilized for fratricide resistance and shown to increase expansion without impairment of cytotoxicity. Gomes-Silva and colleagues showed that in mouse xenograft models, CRISPR-Cas9 ablation of CD7 expression engenders CAR T-cells fratricide resistance, permits robust expansion, and does not compromise target antigen recognition [38]. There is phase I trial of autologous anti-CD7-directed CAR-T cells with a CD28 co-stimulatory domain accruing patients with *r*/*r* T-cell malignancies in children and adults (NCT03690011). 

#### 3.2.2. CD7 off the Shelf CAR

While harvesting T-cells in a patient with B-cell leukemia for CART is generally a safe process for sorting leukemic vs. non-leukemic cells, there is a greater risk of mistakenly selecting a blast in the sorting process for patients with T-ALL and creating a malignant CAR T-cell. To mitigate this risk, one strategy is using autologous or “off the shelf” donor-derived CAR T-cells. This strategy also abrogates challenges with T-cell fitness after collecting T-cells from patients who have recently received cytotoxic chemotherapy. 

Cooper and colleagues created UCART7, a fratricide-resistant, off-the-shelf product of CD7-directed CAR T-cells created with CRISPR-Cas9 editing to disrupt the CD7 and TCRα constant (TRAC) loci. In a study of NGS mice engrafted with T-ALL blasts, treatment with UCART7 donor cells demonstrated tumor clearance without development of GVHD [39]. 

Diorio and colleagues recently reported an alternative approach using cytosine base editing (CBE) genome technology as an alternative to DNA double strand break (DSB)-inducing nucleases or CRISPR-Cas nucleases. Using CBE, this group developed 7CAR8, a CD7-directed allogeneic CART using four simultaneous base edits. They demonstrated that CBE does not impact T-cell proliferation or lead to aberrant DNA damage response or karyotypic abnormalities which can be seen with CRISPR-Cas9 editing. They also found 7CAR8 to be highly efficacious against T-ALL in multiple in vitro and in vivo models. This modality is preferable to DSB editing which can also result in unintended outcomes such as complex genomic rearrangements and chromothripsis [87]. 

Another novel technique for antigen downregulation in CARs is protein expression blockers (PEBLs). PEBLs function by entrapping the protein of interest within the endoplasmic reticulum or Golgi apparatus, preventing cell surface expression. In vivo models of PEBL-generated CD7-directed CAR T-cells injected in xenograft mice demonstrated significant treatment advantage over control mice; however, all of these mice did ultimately relapse [88]. 

A phase I/II clinical trial exploring safety and efficacy of anti-CD7 allogeneic CAR-T (WU-CART-007) in patients with relapsed or refractory T-ALL and T-LBL for patients aged 12 years and older is currently enrolling (NCT04984356).

#### 3.2.3. CD5 CAR

CD5 is a transmembrane glycoprotein expressed on lymphocyte precursors, mature T-cells and a subset of mature B-cells. It is thought to play a role in preventing autoimmunity and recognition of cancer cells [89]. CD5 is present in approximately 80% of T-ALL and T-LBL. Because expression on T-cells is approximately ten times greater than on B-cells [90], a low affinity, high avidity CD5-directed CAR T-cell could potentially spare B-cell aplasia [91]. 

In T-ALL xenograft models, CD5 CAR T-cells initially delayed expansion by 2–3 days due to fratricide; however, proliferation and expansion was subsequently unimpaired. Resistance to fratricide was mediated by: (a) downregulation of CD5 antigen either by masking or internalization, and (b) elevated levels of molecules protecting against cytotoxic effects of perforin/granzyme and Fas/FasL pathways used to trigger apoptosis [92].

There is currently one open clinical trial of anti-CD5 CAR T-cells for the treatment of children and adults with T-ALL and T-LBL (NCT03081910). Initial results from this ongoing study are somewhat promising: 4–8 weeks post CD5 CAR T-cell infusion, four out of nine evaluable patients obtained response, and CR was achieved in three patients. Two of these patients did not undergo transplant and subsequently relapsed at 6 weeks and 7 months post infusion. The third patient was undergoing workup for HSCT at time of published results. Notably, there was no severe toxicity (above Grade 2) and complete T-cell aplasia was not observed [93].

#### 3.2.4. hTERT CAR

Telomeres are nucleoprotein structures that sit at the terminal ends of chromosomes and consist of highly conserved tandem DNA repeats. Telomerase is an enzyme which functions to extend the length of telomeres; after birth its activity is silenced or downregulated in somatic cells. It contains two essential subunits: human telomerase reverse transcriptase (hTERT) and the RNA subunit human telomerase RNA (hTERC). Upregulation of telomerase activity is observed in over 85–90% of human cancers; thus, targeting telomerase is an attractive approach [94]. 

hTERT has been shown to play an important role in oncogenesis through cell immortalization [95]. Using patient-derived xenograft models, Miyazaki and colleagues found that T-cell targeting of hTERT with modification of the T-cell receptor (hTERT-siTCR) led to control of adult T-cell leukemia (ATL) [96]. While hTERT antigen targeting has not yet been studied in pediatrics, it has been shown that pediatric T-ALL cells frequently overexpress hTERT, and thus this may be another option for targeting in the future [97,98]. Off target effects from hTERT targeting remain unknown and caution is warranted. 

#### 3.2.5. CD1a CAR

Sanchez-Martinez and colleagues reported on CD1a-directed CAR T-cells with persistent anti-leukemic activity with in vivo xenograft models with fratricide resistance [99]. This target should be explored further; however, only a minority of pediatric cases with T-ALL express this antigen.

### 3.3. NK CARs

NK cell CARs are an alternative approach which may be particularly beneficial for T-cell malignancies to avoid fratricide, malignant contamination, and GVHD. Whereas αβ CAR T-cells generally have a memory phenotype which can lead to persistence and thus T-cell aplasia, NK CARs are more short-lived. Although this may necessitate multiple NK CAR doses to induce remission, shorter persistence may also limit off-tumor effects. Additionally, NK-CARs may have a lower risk of GVHD and cytokine release syndrome [100]. 

You and colleagues demonstrated that CD7-directed NK cells (CD7-CAR-NK-92MI and dCD7-CAR-NK-92MI) effectively killed T-ALL blast in vitro and in vivo, improving overall survival of mouse xenografts compared to controls [101]. Chen et al. also generated CD3 CAR-NK92 cells with efficacy at killing CD3+ human peripheral T-cell lymphoma and T-ALL cells. Furthermore, they demonstrated enhanced tumor control in T-ALL xenogeneic mouse models [102]. This group also studied CD5-directed CAR NK92 cells and found consistent anti-tumor activity in vitro and in vivo [103]. There is currently one open NK CAR trial for CD5+ *r*/*r* T-cell malignancies lymphoma (NCT05110742).

## 4. Immunotherapy for Peripheral T-Cell Lymphoma

Peripheral T-cell lymphomas are a group of aggressive T-cell malignancies that develop outside of the thymus or bone marrow. The most common PTCL in children is anaplastic large cell lymphoma (ALCL), representing about 10–15% of all pediatric NHL. The majority of pediatric cases of ALCL are ALK-positive and have superior prognosis as compared with ALK negative disease, potentially due to greater chemotherapy responsiveness [104]. Up-front treatment for ALCL is combination chemotherapy. In the relapsed setting, ALK inhibitors can be used for patients with ALK aberrant disease; however, there are fewer options for ALK negative patients, and alternative novel immunotherapies are needed.

### 4.1. Antibody Drug Conjugates

#### CD30: Brentuximab Vedotin 

CD30 is a transmembrane glycoprotein receptor with intracellular, transmembrane and extracellular domains. In healthy cells, it is expressed on a subset of activated T-cells and B-cells and is thought to play a role in immune surveillance and B-cell/T-cell interaction. In malignancies it is consistently overexpressed in HL and ALCL, with variable expression in other lymphomas [105]. Given its relatively restricted expression to tumor cells, CD30 has been an intriguing target for antibodies and CARs given limited risk for off-target toxicity.

Brentuximab vedotin (BV) is a chimeric monoclonal antibody targeting CD30 linked to anti-tubulin agent monomethyl auristatin E (MMAE). When the antibody effectively binds CD30, the drug receptor complex is internalized and leads to MMAE release within the target cell, driving apoptosis [106]. The ECHELON-2 trial was a multinational double-blind randomized trail randomizing patients with ALCL and other CD30-positive PTCLs to receive either CHP (cyclophosphamide, doxorubicin, prednisone) and BV, or standard CHOP (cyclophosphamide, doxorubicin, Oncovin, prednisone). Patients treated with CHP + BV showed superior progression-free survival and significantly longer OS with comparable adverse events. Median progression-free survival was 48.2 months in the brentuximab group and 20.8 months in the standard of care arm (*p* = 0.0110) [107]. 

Given success in adults, a phase I/II study in pediatric patients with relapsed HL or ALCL demonstrated an overall response rate of 53%. Subsequently, a COG phase II study (ANHL12P1) comparing addition of BV to ALCL99 chemotherapy regiment for newly diagnosed ALK-positive pediatric ALCL found superior relapse prevention in the BV arm compared to conventional chemotherapy alone, without additional toxicity [106]. 

There are several open clinical trials utilizing BV. Open phase II clinical trials in adults include: BV alone versus BV in combination with nivolumab (NCT01703949); BV in combination with lenalidomide (NCT03409432); and BV as single agent therapy (NCT03947255). 

### 4.2. Immune Checkpoint Inhibition

#### PD1, CTLA4

The immune system strikes a careful balance of stimulatory and inhibitory signals—also known as immune checkpoints. The programmed cell death protein (PD-1) is induced in the setting of T-cell activation but not expressed in resting T-cells. When PD-1 is activated, it can bind its ligands (PD-L1 and PD-L2), which sends signals to inhibit T-cell receptor-mediated cell proliferation, downregulating the host immune response. Many malignant cells have evolved to express PD-1 ligands on the cell surface to downregulate host immune response by T-cell exhaustion [108]. 

CTLA4 is also an immune checkpoint which acts similarly to PD-1. Following T-cell receptor (TCR) engagement, CTLA4 expression is upregulated, dampening T-cell signaling through competitive inhibition of B7 ligands (B7-1 and B7-2) and thus attenuating T-cell activation [109]. 

Checkpoint inhibitors are being explored for treatment of T-cell malignancies—although thus far responses have been modest. A phase II trial explored efficacy of nivolumab (anti-PD-1 monoclonal antibody) in adult T-cell leukemia-lymphoma (ATLL) (NCT02631746), but unfortunately the trial was halted after three patients had rapid disease amplification after a single dose of nivolumab [110]. The study investigators obtained primary cells from these patients and surprisingly noted clonal expansion after PD-1 blockade of unclear cause [111]. A phase II study also examined pembrolizumab (anti-PD1-antibody) for patients with *r*/*r* T-cell lymphoma. Of 13 patients evaluable for response, overall response rate was 33%. Median progression-free survival was 3.2 months and median overall survival only 10.6 months [112]. Immune checkpoint inhibitors in combination with other immunomodulatory agents may be a more promising avenue. 

### 4.3. CAR T-Cell Therapy 

#### 4.3.1. CD30

As described, CD30 has been targeted with BV and shown good responses but challenges with durability; thus, CD30-directed CAR T-cells are being explored. 

Anti-CD30 CAR T-cells were originally studied in HL and demonstrated a high rate of durable responses [113]. In a phase I trial, Ramos and colleagues examined anti-CD30 CART in patients with *r*/*r* HL or ALCL. They found that of seven patients with relapsed HL, one entered a complete response (CR) lasting more than 2.5 years, one remained in CR for almost 2 years, and three had transient stable disease [114]. Of two patients with ALCL, one patient had a CR lasting 9 months. Notably, no patients seemed to have impairment of viral immunity. Wang and colleagues also tested an anti-CD30 CAR T-cell in 18 patients with *r*/*r* CD30+ lymphoma (17 HL, 1 ALCL) and found mixed responses with a median progression-free survival of 6 months [115]. 

Hucks et al. explored anti-CD30 CART for HL and ALCL in pediatric patients. In a phase II study, five patients with *r*/*r* disease (3 HL, 2 ALCL) were treated with anti-CD30 CAR T-cells following lymphodepleting chemotherapy with bendamustine and fludarabine. One patient with ALK+ ALCL had progressive disease. The other four patients achieved CR and remained in CR 4 to 24 months post infusion [116]. 

There are several ongoing clinical trials examining anti-CD30 CART for CD30+ HL and NHL (NCT02917083, NCT04526834, NCT01316146, NCT03383965). There is also a phase I study utilizing anti-CD30 CAR T-cells genetically modified to co-express CCR4 (NCT03602157).

#### 4.3.2. TRBC1

T-cells express the αβTCR, which interacts with antigens presented by the major histocompatibility complex. The TCR β-chain can either be encoded by TRBC1 gene or TRBC2 gene, and expression of TCRB1 or TCRB2 is typically mutually exclusive. Among healthy CD4-positive and CD8-positive T-cell populations, there is heterogeneity of expression of both receptors; however, among malignant populations, blasts are generally either TRBC1 or TRBC2-positive with homogenous β-chain expression [117]. 

Macocia and colleagues found that in vitro and in vivo anti-TRBC1 CAR T-cells killed healthy and malignant TRBC1+ T-cells, but spared normal TCRB2+ T-cells. Thus, this may represent a targeted approach to preserve some host T-cell immunity without conferring complete T-cell aplasia [117]. A phase I/II clinical trial is currently recruiting patients with *r*/*r* TRBC1 positive T-cell NHL for patients 18 years old and above. (NCT03590574).

#### 4.3.3. CD4

CD4 is a glycoprotein that serves as a co-receptor for the TCR. In healthy tissue, it is expressed in T-helper cells, macrophages, monocytes and dendritic cells. After CD4+ T-cells are activated, they differentiate into effector cells and are important for many host immune responses [118]. 

CD4 is nearly universally expressed on PTCL. A concern of using CD4 as a target antigen, however, is depletion of CD4 helper T-cells and subsequent immunodeficiency. Two preclinical studies have examined CD4-directed CAR T-cells. Pinz et al. found that CD4 CAR T-cells eliminated a CD4+ leukemic cell line in vitro [119]. This same group then demonstrated effective targeting of CD4+ T-ALL cells in murine models. Additionally, they utilized alemtuzumab as a safety switch to deplete the infused CD4-directed CAR T-cells to prevent T-cell aplasia. Five days following alemtuzumab administration, CD4 CAR T-cells were virtually depleted [120]. There is one open phase I trial using CD4-directed CAR T-cells for CD4+ T-ALL and T-cell lymphoma (NCT03829540). 

#### 4.3.4. CD37

CD37 is a transmembrane protein expressed on healthy B-cells and weakly expressed on T-cells and other immune cells. It is thought to regulate B-cell survival and immune response. CD37 is typically expressed in B-cell malignancies; however, it is also present in some cases of PTCL. Given that it is not expressed on T-cells, CD37 CAR T-cells may evade fratricide and T-cell aplasia. In vitro studies have demonstrated activity of anti-CD37 CAR T-cells against PTCL cell lines [121]. These investigators have also combined anti-CD37 CAR T-cells with anti-CD19 CAR T-cells to generate dual-specific CAR T-cells for treatment of B-cell malignancies. This type of dual targeting has yet to be investigated in T-ALL, and to our knowledge there are no open clinical trials targeting CD37 for T-cell malignancies. 

## 5. Conclusions

Immunotherapy for T-cell malignancies has proved more challenging than initially anticipated. Obstacles such as target heterogeneity, fratricide, product contamination, T-cell aplasia and GVHD are present but not insurmountable. Given the heterogeneity of T-cell blasts, a personalized targeting approach may be warranted based on antigenic expression. Novel strategies, including genetically modified CARs to reduce fratricide and off the shelf products for fratricide resistance and faster production times with lower contamination risk, are enhancing options for patients with relapsed and refractory disease. Dual targeting is another creative approach that warrants further investigation. 

In the next decade, immunotherapy will likely save many children with relapsed and refractory T-cell malignancies who were previously untreatable. With thoughtful risk stratification and integration in frontline therapy, these novel approaches may also help mitigate relapse. 

## Figures and Tables

**Table 1:** Target antigen function, expression, therapies, open clinical trials and eligibility.

Target	Function	Healthy Tissue Expression	Targeted Therapy	Open Clinical Trial	Eligible Diseases
CD1a	Lipid antigen presentation, immunity	Early T-cells, Langerhans cells	CD1a/CD 3ε BiTE	NA	
CD4	Antigen recognition	T-cells, macrophages, monocytes, dendritic cells	CD4 CART	NCT03829540	T-cell leukemia and lymphoma
CD5	Prevention of autoimmunity, cancer recognition	T-cells, subset of B-cells (B1 cells)	CD5 CART CD5 NK CAR	NCT03081910 NCT05110742	T-ALL, T-LBL T-cell malignancies
CD7	Humoral and cell mediated immunity	T-cells, NK cells	CD7 CART	NCT03690011 NCT04984356	T-ALL, T-LBL T-ALL, T-LBL
CD25	T-cell proliferation, immune function and immune tolerance	T-cells, B-cells	Basiliximab	N/A	N/A
CD30	Immune surveillance, B-cell and T-cell cross talk	Subsets of T-cells and B-cells	Brentuximab vedotin	NCT01703949 NCT03409432 NCT03947255	PTCL PTCL PTCL
			CD30 CART	NCT02917083 NCT04526834 NCT01316146 NCT03602157	T-cell NHL ALCL T-cell NHL T-cell NHL
CD37	B-cell survival and immune response	B-cells	N/A	N/A	N/A
CD38	Hydrolyzes NAD, important for calcium metabolism	T-cells, B-cells, low level hematopoietic progenitors, endothelial cells, neurons	Daratumumab	NCT05289687 NCT03384654	MRD+ T-ALL T-ALL, T-LBL (pedi/YA)
			Isatuximab	NCT03860844	T-ALL
CD52	Anti-adhesion, co-stimulation CD4+ T-cells	Lymphocytes, monocytes, dendritic cells, epididymis, sperm	Alemtuzumab	NCT03989466	TPML
CD194	Leukocyte homing	T-cells	Mogamulizumab	NCT04848064	T-ALL, T-LBL (adults only)
IL7 Rα	T-cell development, differentiation	T-cells	Anti-IL7rα antibody	N/A	N/A
hTERT	Extension of telomeres	Low or no expression in somatic cells	hTERT-*si*TCR	N/A	N/A
PD1	Immune checkpoint, inhibition of T-cell proliferation	Activated T-cells, NK cells	Nivolumab Pembrolizumab	N/A	N/A
CTLA4/CD152	Immune checkpoint, dampens T-cell signaling	Activated T-cells, NK cells	Ipilimumab Trememilumab	N/A	N/A
TRBC1	Antigen recognition	T-cells	TRBC1 CART	NCT03590574	TRBC1+ T-cell NHL, including ALCL

## Data Availability

Not applicable.
